# Transverse colon volvulus: A case report of an uncommon cause of acute abdomen in pediatrics

**DOI:** 10.1002/ccr3.8828

**Published:** 2024-05-10

**Authors:** Evenildo Martinez Ortega, Dollis De Jesús Rodríguez Ruano, Raed M. Al‐Zoubi, Amani N. Alansari

**Affiliations:** ^1^ Department of Pediatric Surgery Juan M Marquez Children's Hospital Havana Cuba; ^2^ Department of Pediatric Surgery Hamad Medical Corporation Doha Qatar; ^3^ Surgical Research Section, Department of Surgery Hamad Medical Corporation Doha Qatar; ^4^ Department of Biomedical Sciences, QU‐Health, College of Health Sciences Qatar University Doha Qatar

**Keywords:** diagnosis, pediatric patients, surgical emergency, surgical treatment, transverse colon volvulus

## Abstract

Transverse colonic volvulus (TCV) is a serious condition with a mortality rate of up to 33%. It is very rare, especially in children. Despite its rarity, surgeons should have a high index of suspicion and include it in the list of differential diagnoses, especially in patients with developmental delays and associated uncommon syndromes. Resection and anastomosis, whether as a one‐stage or two‐stage procedure, proved to be the best treatment options for children. Since prompt identification and management are vital, this paper presents useful information on the presentation, treatment, and outcome of this case report.

## 1 INTRODUCTION

Colonic volvulus is a rare condition characterized by the obstruction of the colon due to its axial rotation around the mesenteric axis.^1^ Transverse colon volvulus (TCV) is particularly uncommon among the different types of colonic volvulus, especially in the pediatric age group^2^ accounting for nearly 3% of all cases of colonic obstruction^3^ and approximately 3%–5% of all cases of intestinal obstruction.^4^, ^5^ Transverse colonic volvulus has been recorded in less than 100 instances worldwide, mostly in adults and elderly of African descent.^6^ Volvulus mainly affects the sigmoid and cecum, but the transverse colon is seldom impacted.^7^, ^8^

Colonic volvulus involves a “mobile” colon segment with elongated mesentery, termed “sigmoid volvulus (SV), and TCV complex.” The elongated mesentery lets the colon rotate, causing obstruction, and ischemia.^9^ Anatomical factors, such as the transverse colon's short mesentery, predispose to volvulus. Understanding these aspects is crucial for comprehending volvulus' pathogenesis and clinical presentations.^10^ Anatomically, the descending and ascending portions of the colon are fused to the retroperitoneum, whereas the sigmoid and transverse portions are free. Developmental anomalies of fixation, seen in some cases of volvulus and malrotation, are one of the congenital predisposing factors for volvulus. Chronic constipation and high fiber content in the diet result in large volumes of stool and an elongated colon, leading to twisting of the bowel around its mesentery.^11^

The underlying reasons for the increased occurrence of TCV in individuals of the black race remain elusive.^12^ Accumulating evidence from literature documented the high prevalence of colonic volvulus in the Middle East, India, South America, Africa, and Russia where volvulus accounts for about 50% of all cases of colonic obstruction.^13^ The presence of minor mesenteric connections and a greater incidence of extended colon mesentery may enhance the vulnerability of colonic volvulus in those populations.^8^, ^11^ Several risk factors have been identified to have significant implications for the occurrence of colonic volvulus including intestinal malrotation, enlarged colon, abdominal adhesions, and pregnancy. Other contributing factors include a high‐fiber diet, chronic consumption with extensive laxative use, and myopathy.^14^

Transverse colonic volvulus is considered a surgical emergency that prompts timely diagnosis and intervention. The mortality rate has been reported to range from 18%^15^ to 20% of cases due to cardiopulmonary complications^16^ and delay in diagnosis.^17^ Furthermore, the comorbidity score (Elixhauser‐Van Walraven mode comorbidity scores) was higher in patients with transverse colonic volvulus despite their young age compared to sigmoid and cecal volvulus.^15^, ^18^

One aspect that adds significance to this case report is the scarcity of published data on colonic volvulus from Cuba. This case report intends to increase healthcare practitioners' knowledge of TCV, particularly in children. Sharing the experience with this case and reviewing similar cases from the literature facilitates early diagnosis, appropriate management, and optimal outcomes for future patients with TCV.

### 1.1 Case report

**FIGURE 1:**
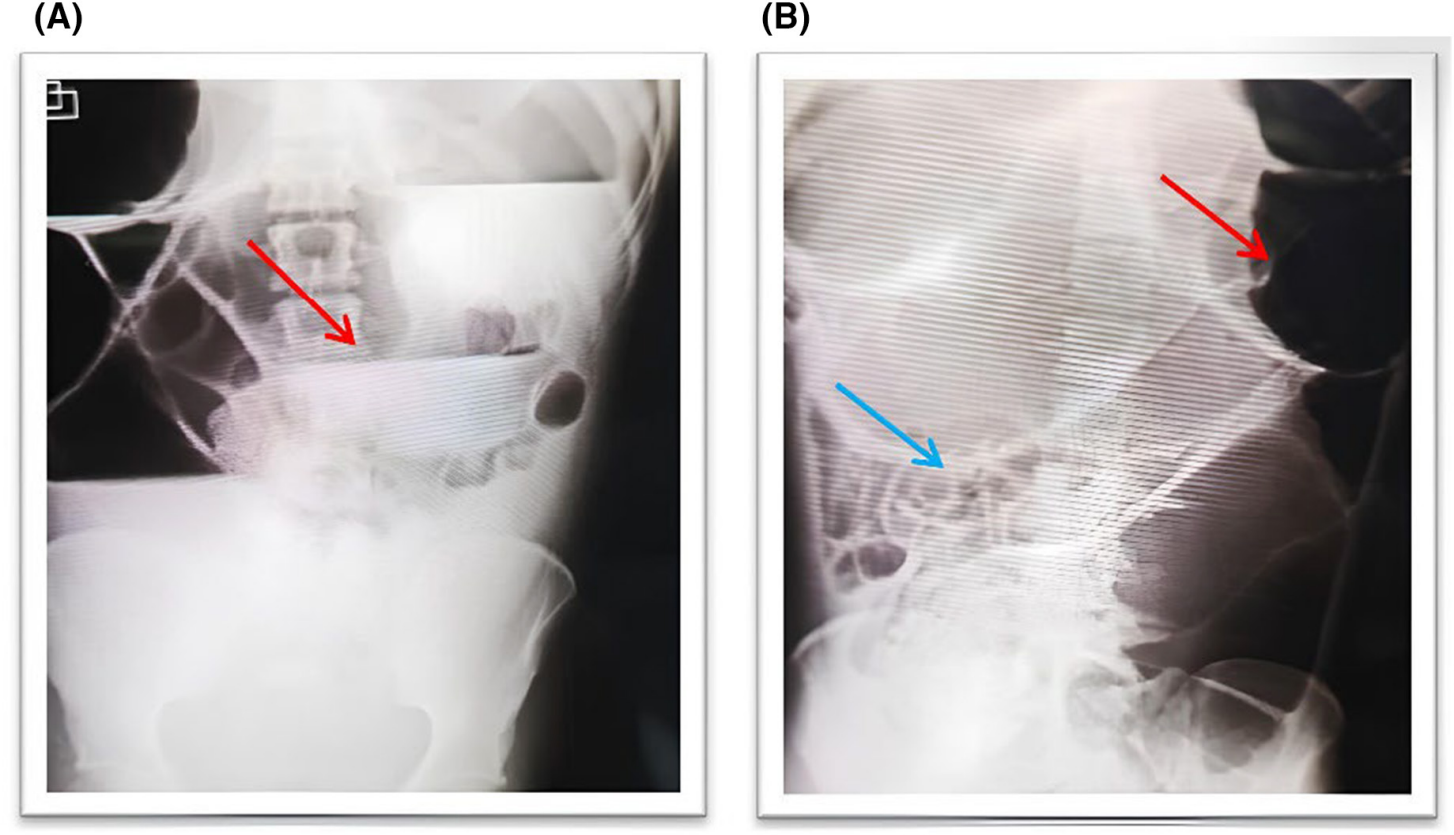
(A) PA (erect) air‐fluid level as a sign of mechanical obstruction; (B) AP (supine) Red arrow shows a dilated colon, and the blue arrow points to the thickened bowel wall.

A 16‐year‐old male with a history of learning difficulties, developmental and mental delay, along with chronic constipation presented to the emergency department with alarming symptoms. He complained of intense cramping abdominal pain and had vomited coffee‐ground material for over 24 h. Upon physical examination, he exhibited signs of dehydration, and significant abdominal distension, particularly in the upper abdomen. Superficial and deep palpation elicited tenderness, and hypertympanism was noted as well. Increased air‐fluid noises were observed during auscultation. A digital rectal examination revealed a rectum filled with stool. Initial blood tests and plain X‐rays were requested. The blood analysis showed a white blood cell count (WBC) of 13.0 10^9^/L (Normal Value 4,5–11,0 × 10^9^/L), hemoglobin (HGB) level of 12.04 g/dL (Normal Value 11–14 g/dL), and platelet count (PLT) of 181.0 10^9^/L (Normal Value 150 a 400 × 10^9^/L). Additionally, the blood gas study indicated the presence of metabolic acidosis. Two views of imaging were taken shown in Figure 1A,B.

### 1.2 Investigations

**FIGURE 2:**
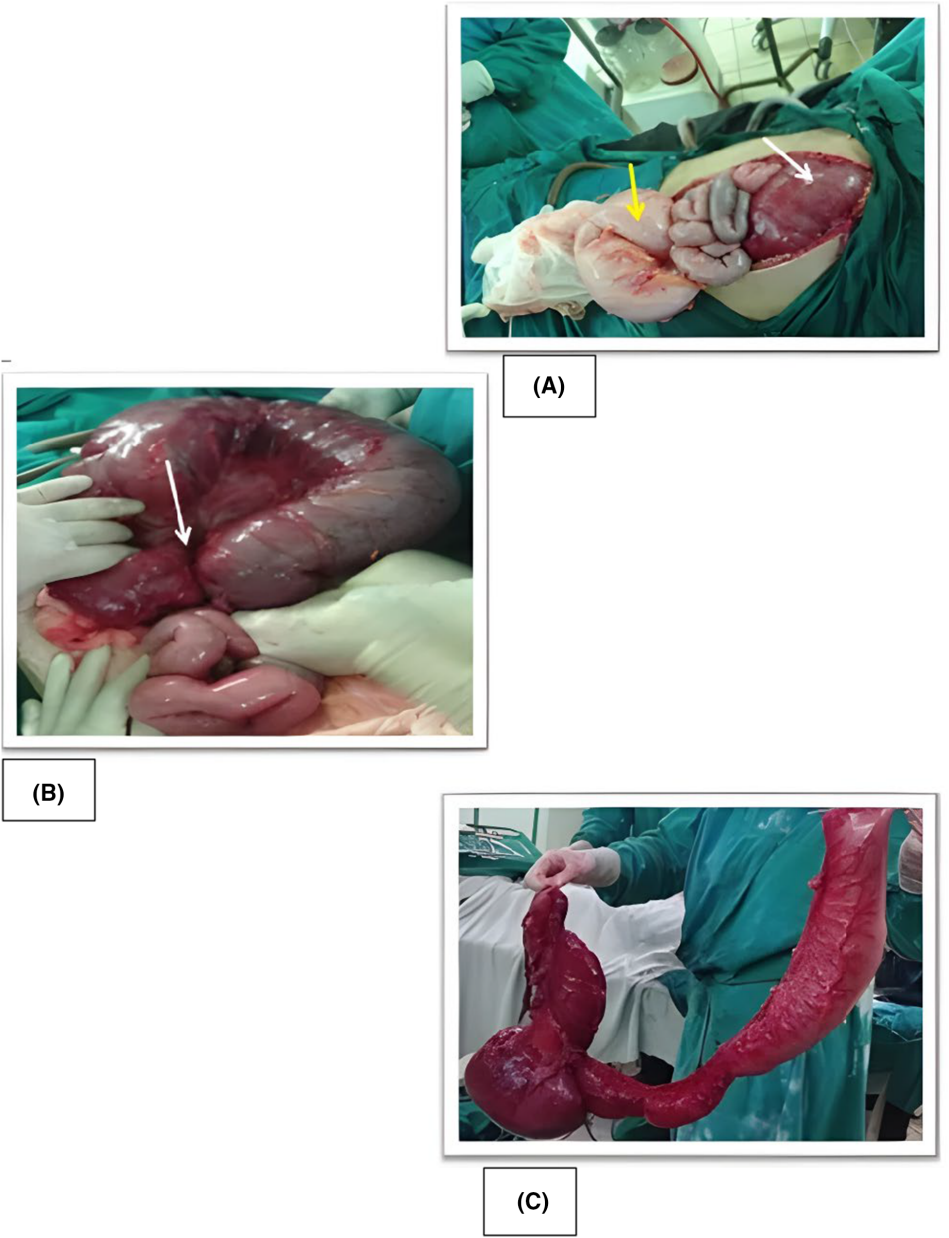
(A) Dilated transverse colon (white arrow), Dilated sigmoid (yellow arrow), small bowel in between; (B) Dilated Ischemic transverse colon. Area of twist (white arrow); (C) Resected dilated transverse colon (150 cm).

The X‐rays revealed marked distension of the large intestine, especially in the upper and central parts of the abdomen, with a thickened bowel wall and an evident air‐fluid level in the transverse colon. Because of the severity of the patient's overall condition, he was admitted to the Intensive Care Unit (ICU) for stabilization before undergoing an emergency laparotomy. Intraoperatively, the diagnosis of TCV was established. Findings are shown in Figure 2A–C:

## 2 SURGICAL PROCEDURE

Initially, fluid resuscitation started along with the appropriate analgesics, and broad‐spectrum antibiotics. Then, the patient was pushed for an emergency laparotomy. Upon exploration, the colon was thoroughly examined, and the volvulus area was identified. The volvulus involves the entire transverse colon, from the upper portion of the ascending colon to almost two‐thirds of the descending colon. The affected segment was found to be vascularly compromised with no sign of peritonitis. The remaining bowel was healthy. Consequently, the decision was made to proceed with a one‐stage procedure rather than two. A total of 150 cm of the volvulus part was resected, and end‐to‐end anastomosis was performed.

## 3 POSTOPERATIVE COURSE

Following the surgery, the patient was transferred to the ICU for close monitoring. He was introduced to liquid feeding 24 h after the procedure, which he tolerated well. He started passing stool after 72 h. His condition remained stable. After spending 4 days in the ICU, the patient's overall health condition improved, and all laboratory investigations indicated a positive recovery.

Afterward, he was transferred to the floor for further observation. The patient was hospitalized for 8 days before being discharged home in stable condition.

A follow‐up in the outpatient department was scheduled after 2 weeks and another after 1 month. In the event of a similar incident, the patient's mother was instructed to seek immediate medical attention. According to the latest follow‐up, the patient appears to be doing well 2 years after the operation.

## 4 DISCUSSION

TCV is a relatively rare cause of bowel obstruction, accounting for only 1%–3% of all colonic volvuli cases.^18^ It is more frequent in tropical/subtropical areas, (13%–42%) in comparison to the USA and Western Europe (5%).^15^, ^20^ SV occurs most frequently at 60%–75%, While TCV is reported least to happen at 1%–4%.^21^ TCV can also occur synchronously with SV, especially among elderly patients and to a lesser extent in young adults.^19^, ^22^

The condition arises when the transverse colon twists upon its mesentery, obstructing the bowel lumen. This type of volvulus is rare due to the short mesentery and the presence of hepatic and splenic attachments that typically prevent extensive twisting.^23^ However, predisposing factors such as previous mobilization of the colonic flexures and chronic dilatation of the colon may contribute to its occurrence.

TCV may be linked to underlying medical disorders that raise the likelihood of volvulus development in specific patient groups.^4^ For instance, a case study by Miličković et al.^24^ detailed a 16‐year‐old child with cerebral palsy with a TCV. In a patient with neurological impairment, chronic constipation was shown to be a risk factor. In our case, delayed mental development and learning difficulties presented a social and medical challenge.

TCV may occur as an unusual complication after SV resection, Chinisaz et al.^25^ report a 73‐year‐old man who suffered from this condition. This displays the importance of closely monitoring postoperative patients and being vigilant for potential complications.

Additionally, TCV can be associated with unusual presentations and complications. Kayiira et al.^26^ reported a case of TCV in a 35‐year‐old man who presented with intestinal obstruction features, right lung collapse, and left mediastinal shift. Point‐of‐care lung ultrasound helped rule out pneumothorax and avoid unnecessary interventions. This case highlights the importance of considering rare presentations and utilizing appropriate diagnostic tools to ensure accurate diagnosis and management.

Treatment of TCV depends on various factors, including the severity of the obstruction and the patient's overall health condition.^27^ Although endoscopic decompression can be effective in treating SV, the primary treatment for TCV is surgery, especially for patients with advanced age and multiple comorbidities, to prevent complications and improve patient outcomes. A retrospective study conducted by Safioleas et al.^7^, ^27^ supports this approach.

TCV management can be challenging, especially in patients with underlying uncommon medical conditions. In a case reported by Younus et al.,^28^ a 22‐year‐old adult with Williams Syndrome underwent a subtotal colectomy with end‐to‐end anastomosis. For this reason, physicians should remain vigilant for gastrointestinal complications that can occur in uncommon syndromic patients to initiate the appropriate treatment as soon as possible.

The choice of surgical treatment in TCV depends on a number of factors which include: the viability of the intestine, the presence of perforation or/and peritonitis, the diameter of the bowel, the length of the mesentery, and the overall stability of the patient.^7^, ^20^

There are two main surgical treatment options for intestinal volvulus: first, endoscopic or operative detorsion with or without colpopexy. However, it is not recommended due to the reported high rate of recurrence. The 2nd option is resection of the volvulus segment with primary anastomosis (one‐stage procedure) or resection with the creation of a stoma followed by the closure of the stoma in 6–8 weeks (two‐stage procedure). Resection and anastomosis are the recommended surgical procedures in the context of TCV with a low rate of recurrence and better outcomes.^4^, ^7^, ^17^

Given our patient's medical history of learning difficulties and the radiological and clinical findings suggesting acute obstructive abdominal, emergency laparotomy was the treatment of choice. The lack of signs of infection and the remaining healthy bowel was encouraging to opt for resection and anastomosis without stoma to avoid unnecessary delay in bowel restoration and enhance recovery.

Bhandari et al.^21^ conducted a retrospective review of Adult patients with colonic volvulus in Nepal. They found that surgical complications were significantly associated with factors such as age over 60, preoperative hypotension (systolic blood pressure below 90 mmHg), and the presence of gangrenous bowel. However, the causal relationship between volvulus and pediatric population factors has yet to be established.

The mortality rate associated with TCV can be significant at 33%. Morice et al.^18^ reported TCV in a 90‐year‐old who had an emergency laparotomy and subtotal colectomy. A postoperative complication resulted in the patient's death 6 days later. Thus, early diagnosis and intervention are essential to improve survival rates among patients with TCV.

Considering all previous scenarios, it is evident that the rarity of such pathology underscores the importance of including it in a patient's differential diagnosis when they present with acute abdominal pain and bowel obstruction, especially in places where colonic volvuli are more common. To minimize mortality and morbidity associated with this condition, early diagnosis, and appropriate intervention are essential.^29^, ^30^

## 5 CONCLUSION

The occurrence of TCV resulting in intestinal obstruction is rare; however, if not promptly addressed, it can lead to life‐threatening consequences. Its atypical clinical manifestations and intricate treatment regimen necessitate vigilant consideration. Surgical intervention remains the definitive choice, especially in medically compromised pediatric cohorts. The confluence of TCV alongside other colonic volvuli can complicate differential diagnosis, underscoring the importance of a comprehensive clinical perspective and robust diagnostic resources. Clinicians can reduce morbidity and mortality by refining treatment strategies for TCV.

## AUTHOR CONTRIBUTIONS

**Evenildo Martinez Ortega:** Conceptualization; data curation; investigation; methodology; writing – original draft. **Dollis De Jesús Rodríguez Ruano:** Conceptualization; data curation; investigation; methodology; writing – original draft. **Raed M. Al‐Zoubi:** Writing – original draft; writing – review and editing. **Amani Nasser Al‐Alansari:** Conceptualization; supervision; writing – original draft; writing – review and editing.

## FUNDING INFORMATION

This research did not receive any specific grant from the public, commercial, or not‐for‐profit funding agencies.

## CONFLICT OF INTEREST STATEMENT

The authors of this manuscript have no conflicts of interest to declare. All co‐authors have seen and agree with the manuscript's contents and there is no financial interest to report.

## ETHICS STATMENT

The patient was informed and agreed that data concerning the case would be submitted for publication. The Medical Research Center and Institutional Review Board (**IRB**) of Juan M Marquez Children's Hospital confirmed the patient's consent, confirmed that data was anonymized, and agreed with publication.

## CONSENT

Written informed consent was obtained from the patient for the publication of this case report and the accompanying images.

## Data Availability

Data will be made available on request.
